# Adjuvant Atezolizumab for Stage III Deficient Mismatch Repair Colon Cancer—Its Impact on Clinical Practice

**DOI:** 10.1002/mco2.70952

**Published:** 2026-08-12

**Authors:** Yun Wu, Shanshan Weng, Ying Yuan

**Affiliations:** ^1^ Department of Medical Oncology Key Laboratory of Cancer Prevention and Intervention Ministry of Education The Second Affiliated Hospital Zhejiang University School of Medicine Hangzhou China; ^2^ Center for Medical Research and Innovation in Digestive System Tumors Ministry of Education Hangzhou China

1

In the ATOMIC phase III trial recently published in The New England Journal of Medicine [1], Sinicrope et al. demonstrated that adding atezolizumab to standard adjuvant chemotherapy (mFOLFOX6) significantly improved disease‐free survival (DFS) in patients with resected stage III deficient mismatch repair (dMMR) colon cancer, providing the first phase III evidence for adjuvant immunotherapy in this molecular subgroup.

In this randomized trial, 712 patients were assigned to receive either mFOLFOX6 alone for 6 months or atezolizumab plus mFOLFOX6 for 6 months followed by atezolizumab monotherapy for an additional 6 months. Notably, 53.9% of enrolled patients had high‐risk disease (T4 and/or N2 tumors). The addition of atezolizumab significantly improved DFS, reducing the risk of recurrence or death by 50%. At a median follow‐up of 40.9 months, the 3‐year DFS rate was 86.3% in the combination arm versus 76.2% in the chemotherapy‐alone arm (hazard ratio [HR], 0.50; 95% confidence interval [CI], 0.35–0.73; *p* < 0.001). The benefit was generally consistent across major clinical subgroups. Overall survival (OS) data remain immature; at the first analysis, no significant between‐group difference in OS was observed (HR, 0.90; 95% CI, 0.55–1.47). Future OS analyses may be difficult to interpret because post‐recurrence checkpoint inhibitor therapy was common in the chemotherapy‐alone arm. Importantly, the National Comprehensive Cancer Network (NCCN) Guidelines for Colon Cancer (Version 1.2026) list FOLFOX plus atezolizumab and CAPEOX plus atezolizumab as preferred adjuvant treatment options for stage III dMMR/microsatellite instability‐high (MSI‐H) colon cancer [2].

Why does ATOMIC matter so much? dMMR colon cancer represents a biologically distinct and highly immunogenic subtype, characterized by defective mismatch repair, high tumor mutational burden (TMB), dense lymphocytic infiltration, and an immune “hot” microenvironment. While these features provide a strong biological rationale for immune checkpoint blockade, the clinical management of stage III disease has remained based on oxaliplatin‐containing chemotherapy, regardless of mismatch repair status. In this context, ATOMIC supports the incorporation of immunotherapy into adjuvant treatment for stage III dMMR colon cancer.

ATOMIC is also important in the broader treatment landscape of dMMR/high microsatellite instability colorectal cancer (dMMR/MSI‐H CRC). Immunotherapy has already transformed the treatment of metastatic dMMR/MSI‐H CRC, with KEYNOTE‐177 establishing pembrolizumab as a first‐line standard of care and CheckMate‐8HW further supporting dual‐agent immunotherapy (nivolumab and ipilimumab) as a chemotherapy‐free first‐line option in this molecular subgroup [3]. In localized disease, neoadjuvant studies such as NICHE‐2 have also demonstrated remarkable pathological responses after short‐course immunotherapy [4]. Despite these advances in metastatic and neoadjuvant settings, the role of immunotherapy in the adjuvant setting remained uncertain until recently. ATOMIC therefore extends the benefit of immunotherapy into the postoperative treatment of stage III dMMR colon cancer.

ATOMIC has established a new benchmark for adjuvant therapy in stage III dMMR colon cancer, but whether chemotherapy can be omitted remains uncertain. In metastatic disease, studies such as KEYNOTE‐177 and CheckMate‐8HW have already established chemotherapy‐free immunotherapy strategies in dMMR/MSI‐H CRC [3]. Although ATOMIC confirmed the benefit of combining chemotherapy with programmed death ligand 1 (PD‐L1) inhibitors, the necessity of the chemotherapy backbone in all patients remains unclear. Notably, patients receiving ≤6 cycles of mFOLFOX6 plus atezolizumab did not appear to derive benefit in exploratory analyses, although the number of events was small. These findings suggest that the role of chemotherapy de‐escalation remains uncertain. As summarized in Table 1, several ongoing trials, including PACE and AZUR‐2, will further clarify whether chemotherapy‐free or perioperative immunotherapy strategies can improve outcomes in dMMR/MSI‐H colon cancer.

**TABLE 1:** Ongoing clinical trials exploring adjuvant immunotherapy in deficient mismatch repair (dMMR) colorectal cancer.

Study name/ NCT number	Region	Phase	Study design	Sample size	Disease stage	Experimental treatment	Control treatment	Primary endpoint	Secondary endpoint	Status
ATOMIC/ NCT02912559	Worldwide	III	Randomized controlled	712	Stage III dMMR colon cancer	Atezolizumab 12 months + mFOLFOX6	mFOLFOX6	DFS	OS and AEs	Active, not recruiting
POLEM/ NCT03827044	United Kingdom	III	Randomized controlled	402	Stage III MSI‐H or POLE‐mutant colon cancer	5‐FU‐based chemotherapy followed by avelumab (6 months)	5‐FU‐based chemotherapy	3‐year DFS	OS, QoL, and AEs	Terminated
SMALLER/ NCT06520683	China	III	Randomized controlled	180	High‐risk stage II dMMR/MSI‐H colorectal cancer	Tislelizumab for two cycles + SOC	SOC	DFS	OS and AEs	Recruiting
PACE/ NCT05236972	China	III	Randomized controlled	323	Stage III dMMR/MSI‐H colorectal cancer	Sintilimab alone 6 months	CapeOX	3‐year DFS	5‐year OS	Recruiting
AZUR‐2/ NCT05855200	Worldwide	III	Randomized controlled	711	T4N0 or Stage III dMMR/MSI‐H colon cancer	Dostarlimab (pre for four cycles and post‐surgery for six cycles)	SOC (FOLFOX/CapeOX)	EFS	OS, pathological response, AEs	Recruiting
ANTONIO/ NCT05118724	Germany	II	Single‐arm	80	High‐risk stage II and stage III dMMR/MSI‐H colorectal cancer ineligible for oxaliplatin	Atezolizumab 6 months	——	3‐year DFS	1‐, 2‐, and 5‐year DFS; 1‐, 2‐, 3‐, and 5‐year OS	Active, not recruiting
TJMUCH‐GI‐CRC01/ NCT04969029	China	II	Randomized controlled	30	High‐risk stage II and stage III MSI‐H or POLE‐mutant colon cancer	Tislelizumab 12 months	CapeOX	3‐year DFS	——	Unknown status
Wfeng‐001/ NCT05231850	China	II	Single‐arm	70	High‐risk stage II and stage III dMMR/MSI‐H colorectal cancer	Tislelizumab 12 months	——	3‐year DFS	5‐year OS and AEs	Not yet recruiting

Clinical trial details were obtained from ClinicalTrials.gov.

Abbreviations: 5‐FU, 5‐fluorouracil; AEs, adverse events; CapeOX, capecitabine and oxaliplatin; DFS, disease‐free survival; dMMR, deficient mismatch repair; EFS, event‐free survival; FOLFOX, folinic acid, fluorouracil, and oxaliplatin; mFOLFOX6, modified fluorouracil, leucovorin, and oxaliplatin; MSI‐H, microsatellite instability‐high; NCT, ClinicalTrials.gov identifier; OS, overall survival; QoL, quality of life; SOC, standard of care.

The optimal sequencing of immunotherapy is also undefined. In the phase II NICHE‐2 trial, 115 patients with locally advanced dMMR CRC (64% cT4) achieved a remarkable pathological complete response rate of 68% and a 3‐year DFS rate of 100% after only 6 weeks of preoperative nivolumab plus ipilimumab [4]. By contrast, ATOMIC reported a 3‐year DFS rate of 86.3% with a postoperative regimen of 6 months of chemotherapy plus 12 months of atezolizumab. Although these results cannot be directly compared because of differences in design, stage, and endpoints, they raise an important question: should immunotherapy be delivered before surgery, after surgery, or in a perioperative sequence? An additional challenge is that radiographic preoperative staging may be less accurate in dMMR colon cancer than in proficient MMR tumors, raising the possibility of overstaging and overtreatment in neoadjuvant strategies. The ongoing AZUR‐2 study, evaluating perioperative dostarlimab versus standard adjuvant chemotherapy, may provide further insight.

More refined patient stratification is needed. In the ATOMIC trial, 13.6% of patients in the combination arm experienced recurrence within three years, indicating that not all dMMR tumors respond uniformly to immunotherapy. Conversely, 77% of patients in the chemotherapy‐only arm remained disease‐free, suggesting that a subset may have a favorable prognosis and could potentially forgo immunotherapy. These findings underscore the need for predictive biomarkers, such as TMB, tumor microenvironment profiles, or circulating tumor DNA, to better identify patients most likely to benefit from adjuvant immunotherapy. In addition, the role of immunotherapy in high‐risk stage II dMMR colon cancer remains uncertain. Notably, NCCN guidance evolved between Versions 4.2025 and 1.2026, reflecting continued caution regarding extrapolation beyond the stage III population directly studied in ATOMIC, given the lack of direct randomized evidence and the uncertain incremental benefit of perioperative systemic therapy in stage II disease, raising concern about overtreatment. Ongoing trials such as ANTONIO and Wfeng‐001 may help define the role of immunotherapy in this setting.

Questions also remain regarding toxicity, treatment duration, and the broader generalizability of these findings. In ATOMIC, grade 3–4 adverse events were more frequent in the atezolizumab arm than in the chemotherapy‐alone arm (84.1% vs. 71.9%), although the overall safety profile was generally consistent with the known toxicities of mFOLFOX6 and atezolizumab. Suspected immune‐related adverse events such as hypothyroidism, hyperglycemia, colitis, and rash were more common with atezolizumab, but no clear increase in clinically meaningful grade 3–4 immune‐related toxicities was observed. Grade 5 adverse events occurred in 6 patients in the atezolizumab arm and 2 patients in the chemotherapy‐alone arm; importantly, only 2 deaths in the atezolizumab arm were considered treatment‐related in the original report. These findings highlight the need to balance efficacy with tolerability in the adjuvant setting. Patient‐reported outcomes from ATOMIC were subsequently reported at ASCO 2026 and showed no clinically meaningful long‐term deterioration in quality of life, although modest early declines during active treatment, particularly in fatigue and treatment burden, were generally small and transient [5]. The 1‐year duration of atezolizumab used in ATOMIC is largely empirical, and whether shorter durations can preserve efficacy while reducing toxicity and treatment burden remains unknown. In addition, because more than 90% of participants in ATOMIC were White or Black, additional data from more diverse populations, including Asian cohorts enrolled in studies such as PACE and AZUR‐2, will be important for broader clinical application.

In summary, ATOMIC demonstrates that the addition of PD‐L1 inhibitors to standard adjuvant chemotherapy significantly improves DFS in stage III dMMR colon cancer. These findings support adjuvant immunotherapy as a new postoperative treatment strategy in this molecular subgroup. At the same time, several important questions remain, including whether chemotherapy can be omitted, how immunotherapy should be sequenced, how long immunotherapy should be continued, and which patients are most likely to benefit. Ongoing clinical trials and biomarker‐driven studies will be important for refining the role of adjuvant immunotherapy in this setting.

## Author Contributions

Y. W. conceived and drafted the manuscript. Y. Y. and S. W. provided critical comments and substantially revised the manuscript. All authors have read and approved the final manuscript.

## Conflicts of Interest

The authors declare no conflicts of interest.

## Funding

Not applicable.

## Ethics Statement

Not applicable.

## Data Availability

Not applicable.
